# Vulvovaginitis in pregnant women

**DOI:** 10.61622/rbgo/2024FPS03

**Published:** 2024-04-02

**Authors:** Geraldo Duarte, Iara Moreno Linhares, Regis Kreitchmann, Andréa da Rocha Tristão, Evelyn Traina, Ivete Canti, Marcos Takimura, Joelma Queiroz Andrade

**Affiliations:** Faculdade de Medicina de Ribeirão Preto Universidade de São Paulo Ribeirão Preto SP Brazil Faculdade de Medicina de Ribeirão Preto, Universidade de São Paulo, Ribeirão Preto, SP, Brazil.; Faculdade de Medicina Universidade de São Paulo São Paulo SP Brazil Faculdade de Medicina, Universidade de São Paulo, São Paulo, SP, Brazil.; Universidade Federal de Ciências da Saúde de Porto Alegre Porto Alegre RS Brazil Universidade Federal de Ciências da Saúde de Porto Alegre, Porto Alegre, RS, Brazil.; Faculdade de Medicina de Botucatu Universidade Estadual Paulista Botucatu SP Brazil Faculdade de Medicina de Botucatu, Universidade Estadual Paulista, Botucatu, SP, Brazil.; Escola Paulista de Medicina Universidade Federal de São Paulo São Paulo SP Brazil Escola Paulista de Medicina, Universidade Federal de São Paulo, São Paulo, SP, Brazil.; Universidade Federal do Rio Grande do Sul Porto Alegre RS Brazil Universidade Federal do Rio Grande do Sul, Porto Alegre, RS, Brazil.; Universidade Positivo Curitiba PR Brazil Universidade Positivo, Curitiba, PR, Brazil; Faculdade de Medicina Universidade de São Paulo São Paulo SP Brazil Faculdade de Medicina, Universidade de São Paulo, São Paulo, SP, Brazil.

## Abstract

• The balanced vaginal microbiome is the main factor defending the vaginal environment against infections. Lactobacilli play a key role in this regard, maintaining the vaginal pH within the normal range (3.8 to 4.5).

•Hormonal and immune adaptations resulting from pregnancy influence changes in the vaginal microbiome during pregnancy.

•An altered vaginal microbiome predisposes to human immunodeficiency virus (HIV) infection.

•Bacterial vaginosis is the main clinical expression of an imbalanced vaginal microbiome.

•Vulvovaginal candidiasis depends more on the host’s conditions than on the etiological agent.

•*Trichomonas vaginalis* is a protozoan transmitted during sexual intercourse.

•The use of probiotics is not approved for use in pregnant women.

## Recommendations

- All complaints of changes in the vaginal contents reported by the pregnant woman must be evaluated to rule out both the changes in the vaginal microbiome and other genital infections.
- Microscopic examination for direct or Gram-stained examination should always be carried out when suspicious changes of infections are detected in the vaginal contents.
- The Amsel’s criteria and staining of the vaginal content smear according to Nugent criteria are used in the diagnosis of bacterial vaginosis in our country.
- The use of metronidazole for seven days is the preferred treatment of bacterial vaginosis in pregnant women. Although oral and vaginal treatments are efficient, oral treatment for seven days is preferred. If there is any impediment to the use of metronidazole, oral clindamycin is also a more commonly used option.
- The wet mount vaginal swab of the vaginal contents is indicated to complement the clinical examination in the diagnosis of vulvovaginal candidiasis. Culture and antifungigrams are only indicated in relapses.
- Vulvovaginal candidiasis in pregnant women must be treated with imidazole derivatives vaginally for seven days, as oral antifungal medications are not approved for use in pregnant women.
- The wet mount vaginal swab of the vaginal contents is the main test to confirm the diagnosis of trichomoniasis.
- The choice for the treatment of vaginal trichomoniasis is oral metronidazole for seven days. Single-dose oral treatment or vaginal treatment produce results with higher failure rates.
- The best strategy must be established in each service, aiming to include the partner in antenatal care.

## Background

Vulvovaginitis is recognized as an extremely relevant clinical event for many reasons; its frequency, clinical manifestations, psychological repercussions, negative influence on sexuality, facilitation of the spread of sexually transmitted infections, possibility of sequelae and deleterious effects on the gestational prognosis. Despite advances in diagnostic methods and the availability of medications, vulvovaginitis continues to challenge obstetrics professionals, particularly in recurrent episodes.^(1,2)^

Considering the defense system of the vaginal environment against infections, differences are observed between women, some with a more stable balance while others present greater vulnerability.^(3)^ In general, systemic and genital changes during pregnancy make it a more likely period to acquire these infections, indirectly indicating that pregnancy would make women more vulnerable to agents of sexual transmission.^(4)^ Adaptations and modifications of the immune system (humoral and cellular) associated with hormonal changes seen during pregnancy could ultimately account for the changes in the vaginal microbiota that result in this greater vulnerability. As a result of these changes, in theory, the local defense mechanism will be impaired in all pregnant women. However, in practice, some women appear to be able to equalize these changes without the microbiome being altered to the point of predisposing to the appearance of vulvovaginitis.^(5,6)^ In addition to these physiological changes, the anatomical changes resulting from water retention and compression of lymphatic vessels increase vaginal exudation, directly influencing the volume and physical characteristics of vaginal contents. This detail can make it hard to establish the differential diagnosis between genital infection and the physiological increase in vaginal contents resulting from the aforementioned adaptations.^(7)^

During pregnancy, in addition to maternal repercussions, vulvovaginitis is associated with unfavorable obstetric outcomes such as preterm labor, premature rupture of membranes, low birth weight and puerperal infection.^(8)^ Note that preterm birth complications represent the first cause of neonatal mortality, corresponding to approximately 35% of the 3.1 million neonatal deaths occurring annually in the world, and genital tract infections have a protagonist role in this situation.^(9)^

Although some studies in the literature present controversial results regarding changes in the genital microbiome and unfavorable gestational outcomes, such studies were developed using simple methods of identifying microorganisms, such as bacterioscopy and/or culture, which have limitations in their results. More recent research using gene amplification techniques to identify microorganisms that make up the vaginal microbiome during pregnancy have demonstrated the association between the presence of protective lactobacillary flora, mainly of *Lactobacillus crispatus*^,^ with favorable gestational outcomes and that changes in the flora are associated with an increased rate of shortening of the uterine cervix and the occurrence of preterm birth.^(10)^

Undoubtedly, preventive care and adequate diagnosis and treatment of vulvovaginitis and cervicitis during pregnancy are extremely important, contribute to maternal well-being and prevent unfavorable gestational outcomes for the maternal-fetal binomial.^(4)^

## How important is it to know the role of the vaginal microbiome in the context of vulvovaginitis?

The first studies on the vaginal microbiota were carried out in the 19^th^ century, when Döderlein’s *Lactobacillus* were described in the vaginal contents of puerperal women without infectious complications, as well as their absence in women with postpartum infection. Over time, science has demonstrated that knowledge of the vaginal microbiome and its changes is fundamental not only for diagnosis and treatment, but also for measuring the balance of the normal microbiome and its possible deviations. This was possible with the development of microscopy techniques, specific microbiological cultures and molecular biology, which made it possible to identify new components of the vaginal microbiota and the great diversity of aerobic, anaerobic and microaerophilic microorganisms that are part of this microbiological environment.^(10,11)^

As stated, the great diversity of the vaginal microbiome requires a refined balance, allowing the presence of Gram-positive, Gram-negative, aerobic and anaerobic germs in the vagina of healthy women of reproductive age. *Lactobacillus* and different species of cocci (*Staphylococcus aureus*, group B *Staphylococcus*, *Streptococcus* sp., *Peptococcus* and *Peptostreptococcus*), bacilli/bacteria (*Gardnerella vaginalis*, *Escherichia coli*, *Bacteroides* spp., *Prevotella* spp., *Diphteroides*, *Propionibacterium*, *Clostridium* spp. and *Fusobacteria*), mycoplasmas and fungi (*Candida albicans*) stand out by frequency, and the predominant colonization is represented by *Lactobacillus*.^(12)^

Vaginal colonization by *Lactobacillus* is influenced by estrogenic action, therefore its concentration changes according to the different stages of a woman’s life. It changes during pregnancy according to the gestational age.^(13)^ In female newborns, who acquired transplacental estrogen, colonization is abundant then, it reduces during childhood until menarche. The concentration of these microorganisms increases during reproductive life and particularly in pregnancy.^(14)^

The maintenance of an acidic vaginal pH by *Lactobacillus* is essential for maintaining the balanced vaginal microbiome (the normal pH range is 3.8 to 4.5). They produce bacteriocins that prevent the proliferation of pathogenic bacteria at this site and also function as an active component of local defense mechanisms, promoting the activation of auxiliary lymphocytes.^(15)^

More recently, culture media-independent techniques have been developed for the identification of bacteria, which have revolutionized the study of microorganisms. Through gene amplification, these techniques allow the study of a fragment of the 16S rRNA of the DNA of the bacterial genome. Thus, it was possible to identify a high number of bacterial clones that were previously unknown in the vaginal fluid of healthy women and women with genital infections.^(16)^ Although such techniques are more complex and expensive than those using only culture (hence they are more used in research), the contribution of this new knowledge is undoubtedly extremely important for better understanding the pathophysiological processes affecting the genital tract.

The more detailed identification of the bacterial composition of the vagina and of several species of *Lactobacillus*, in addition to the already known *Lactobacillus acidophilus*, as the main constituents of healthy vaginal flora was an important advance made possible by molecular biology. Four species of *Lactobacillus* predominate in the vagina of women of reproductive age: *Lactobacillus crispatus*, *Lactobacillus inners*, *Lactobacillus jensenii* and *Lactobacillus gasseri*. For reasons not yet known, the predominance of one of the species is accompanied by the scarcity or absence of others.^(11,16)^ There are also several differences in the properties between *Lactobacillus crispatus* and *Lactobacillus inners*; the predominance of *Lactobacillus crispatus* has been associated with reproductive success and that of *Lactobacillus inners*, with the greater probability of adverse events during pregnancy.^(17,18)^

## Can endocrine and immune adaptations during pregnancy influence the occurrence of vulvovaginitis?

The increased production of hormones by the placenta promotes a series of changes in pregnant women’s immunity. In vitro studies have demonstrated that steroid hormones depress several aspects of cellular immunity in experimental models, including inhibiting graft rejection and suppressing lymphocyte activation. Chorionic gonadotropin produced by the trophoblast has minimal action in suppressing cellular immunity, and physiological levels of alpha-fetoprotein secreted by the fetal liver into the amniotic fluid can depress T lymphocyte proliferation responses. Although there is no doubt about the depression of cellular immunity during pregnancy, there is no consensus on the quantitative variation, distribution and reactivity of such cells during this period. Some authors have suggested depletion of CD4 and increase in CD8, while others claim that the activity of natural killer cells is deficient. The immune response undergoes an important transformation in pregnancy in order to reduce inflammatory activity, with a change in the activity of Th1 (pro-inflammatory) cells to Th2 (anti-inflammatory). This transformation from a predominance of the Th1 to Th2 immune response is characterized by a decrease in the production of cytokines, such as IL-2 and interferon gamma (INFγ), and an increase in IL-4. The action of complement is also increased during pregnancy. Regarding B cell immunity (humoral immunity), no changes were demonstrated in the pregnancy period.^(19)^

As the fetus is genetically different from the mother, adaptations of both humoral and cellular immunity are necessary to avoid the occurrence of rejection. There is evidence that chemotactic activity and adherence capacity of polymorphonuclear leukocytes reduce from the second trimester onwards. These biological events could explain the lower prevalence of autoimmune diseases and the greater susceptibility of pregnant women to certain infections. In general, humoral immunity in pregnant women remains unchanged. Considering cellular immunity, some aspects are preserved (such as delayed immunity reactions), but its selective aspects need to be suppressed for the preservation of the fetus and pregnancy. As previously stated, knowledge about immunity during pregnancy is still incomplete.^(19)^

## Does the vaginal microbiome undergo any adaptation during pregnancy?

Among the various factors acting on pregnant women’s vaginal microbiome, endocrine and immune system modifications stand out, and these promote extremely relevant changes to the reproductive process.^(15)^ Thus, the immunity of the female genital tract performs important functions: combat of invasive exogenous microorganisms and tolerance to endogenous microflora present in the genital tract.^(20)^

Although the increase in estrogen and progesterone influences the vaginal microbiome, the exact roles of these hormones on vaginal immunity have not yet been definitively demonstrated.^(21,22)^ During pregnancy, the vaginal microbiome undergoes changes in order to establish greater stability, with lower variability of microorganisms and higher concentration of *Lactobacillus* species.^(23)^ This is probably due to a greater production of estrogen that induces an increase of glycogen in vaginal epithelial cells, which is metabolized into lactic acid, with a consequent reduction in vaginal pH. The reduction in vaginal pH and the increase in exudation from the vaginal walls protect the vagina against pathogenic microorganisms.^(20)^ However, if this fine balance is broken, the reduction in pH predisposes to the appearance of fungal infections and cytolytic vaginosis.

As previously mentioned, the complex adaptations and changes in maternal immunity during pregnancy protect the fetus and mother against infections, while at the same time prevent fetal rejection by the maternal organism. The vaginal microbiome interferes with the modulation of the immune system and is also affected by it, resulting in greater tolerance to microorganisms. This interrelationship involves an increase in anti-inflammatory cytokines and the initiation of tolerance to endotoxins, with a consequent reduction in the immune response, thereby favoring the adequate development of pregnancy.^(23)^ In turn, changes in the healthy microbiota can, on the contrary, lead to the increase in inflammatory cytokines, with exacerbation of the immune response and consequent induction of a sequence of inflammatory events that can culminate in preterm birth.^(24)^ By altering the vaginal microbiome, vulvovaginitis and cervicitis can result in unfavorable gestational outcomes with implications for both the maternal organism and the gestational prognosis.^(1)^ However, a compromised gestational prognosis is not always observed.^(25)^

## How to approach pregnant women with bacterial vaginosis?

Bacterial vaginosis (BV) is considered the most common among vaginal dysbiosis (changes in the vaginal microbiota), with a global prevalence of up to 30% among women of reproductive age.^(26)^ It is characterized by the replacement of *Lactobacillus* spp. by other bacterial species, most of which are anaerobic.^(27)^

Recent studies have identified the possibility of formation of a polymicrobial biofilm adherent to vaginal epithelial cells in women with BV, absent in most healthy controls. The initial event resulting in this adverse change in the vaginal microbiota is not completely elucidated and has been a significant impediment both to understanding the pathogenesis of this common condition and to optimizing prevention, as none of the proposed treatments to combat it are cleared for use in pregnant women. This biofilm is believed to be one of the main factors explaining treatment failures. Without elimination of biofilm, therapeutic interventions tend to fail. Likewise, maintaining the biofilm would be linked to BV relapses.^(28)^

The presence of BV is associated with a greater likelihood of acquiring and transmitting sexually transmitted agents, such as *Chlamydia trachomatis* and *Neisseria gonorrhoeae*, and there is consistent evidence that it also facilitates infection with the human immunodeficiency virus (HIV).^(29)^

When present, particularly in early pregnancy, BV has been associated with an increased risk of miscarriage, preterm birth, low birth weight, need for advanced life support (neonatal), premature rupture of membranes and intrauterine infection.^(8,30)^ However, the results related to the poor gestational prognosis are contradictory and not confirmed in important publications.^(31)^ Certainly the host’s immune factors with the individual variations and the specific virulence of each microbial strain involved in the particular genesis of that case of BV in pregnant women appear to influence the occurrence of adverse events and complications.^(32)^

The high rate of asymptomatic women carrying BV represents a problem in clinical practice, mainly due to its association with other infections or even other changes in the vaginal microbiome.^(1)^ Since there are no protocols that recommend universal screening of the obstetric population during antenatal care, the best imaginable scenario would be that women planning to become pregnant have a balanced vaginal microbiota, dominated by *Lactobacillus* spp.^(27,33)^ For this, an adequate and easy-to-perform assessment with direct microscopic examinations of the vaginal contents would be necessary.

The clinical diagnosis of BV is based on the complaint (unpleasant odor and white fluid discharge) and gynecological examination. Examination with a speculum allows confirming the presence of whitish or grayish vaginal contents with a homogeneous appearance, generally in small quantities. Even in cases of abundant vaginal contents, there are no signs of inflammation on the vaginal walls and cervix. It is important to remember that pregnancy changes can alter the appearance of the physiological vaginal content, which will be more fluid and generally more abundant.^(4,27)^

Undoubtedly, the most common complaint in BV is the unpleasant odor (of spoiled fish), which worsens after menstruation and after sexual activity with ejaculate in the vaginal cul-de-sac. However, around 50% of women with this dysbiosis do not present symptoms, which constitutes a challenge for diagnosis.^(33)^ It is known that BV is not an isolated cause of dysuria, dyspareunia, pruritus or signs of vaginal inflammation such as edema or erythema.^(4)^

The clinical diagnosis of BV can be confirmed by the presence of at least three of the four criteria established by Amsel et al.,^(34)^ as follows: 1) grayish-white homogeneous vaginal discharge; 2) vaginal pH ≥ 4.5; 3) positive amine test; and 4) presence of clue cells (also known as “key cells” or “guide cells” for diagnosis) in the direct microscopic examination of the vaginal contents.

Considering the auxiliary resources for the diagnosis of BV, remember to measure the vaginal pH, which is above 4.5.^(35)^ The strip must be suitable for measuring vaginal pH, normally ranging from 3 to 8. The pH is elevated due to the marked depletion or absence of *Lactobacillus* spp., which are responsible for the production of lactic acid in the vaginal environment. The vaginal pH is measured by placing an indicator strip in contact with the vaginal contents of the lateral wall of the vagina for approximately 30 seconds and comparing it with a color scale on the tape packaging itself. It is important to avoid contact with the uterine cervix, as cervical mucus is alkaline and can alter pH measurements. The Whiff test (also known as the amine test or smell test) is useful in diagnosis. It is performed by placing a drop of vaginal contents on a glass slide, adding a drop of 10% potassium hydroxide solution. The test is considered positive when volatile bioamines with a foul odor, characteristic of BV, are released. This odor occurs because the anaerobic bacteria present in BV produce diamines, which volatilize when in contact with an alkaline solution, giving off a foul odor (putrescine, cadaverine and trimethylamine). An in vivo example of this biochemical detail is what occurs when semen (alkaline) comes into contact with the vaginal contents of a pregnant woman with BV during sexual intercourse with ejaculate in the vaginal cul-de-sac, releasing an unpleasant smell.^(4)^

The criteria of Amsel et al.^(34)^ present lower sensitivity than the method based on Gram staining of vaginal smears and quantified according to the criteria and scores proposed by Nugent et al. (1991)^(36)^ for the laboratory diagnosis of BV. To date, the analysis of vaginal smears stained by the Gram method and evaluated according to the 10-point score by Nugent et al.^(36)^ is considered the gold standard for this diagnosis. This score is based on the attribution of points according to the semi-quantification of bacterial morphotypes present in the slide smears and classifies the microbiota as: type I (scores 0 to 3), considered normal; type II (scores 4 to 6), considered intermediate; and type III (scores 7 to 10), which indicates a diagnosis of BV (Chart 1).^(36)^ The criteria for quantifying bacteria in the smear were adapted and published by Carvalho et al. (2021)^(37)^ (Chart 2).

**Chart 1:** Method for evaluating vaginal smears stained by the Gram method in the diagnosis of bacterial vaginosis

Bacterial morphology	Points by morphological type				
	0	1+	2+	3+	4+
Large Gram Bacillus (+) (Lactobacilli)	4	3	2	1	0
Small Gram Bacillus (-)	0	1	2	3	4
Curved Gram Bacillus (-)	0	1 or 2	3 or 4	2	2

Source: Modified from Nugent et al. (1991).^(36)^

**Chart 2:** Quantification of bacteria on slides

0: Absence of bacteria 1: <1 bacterial morphotype present 2: 1 -5 bacterial morphotypes present 3: 6 - 30 bacterial morphotypes present 4: 30 or more bacterial morphotypes present

Source: Carvalho et al. (2021).^(37)^

It is already well established that BV is a polymicrobial dysbiosis of the vaginal microbiota and its composition can vary between different cases. Many species of microorganisms have already been identified as associated with BV by using culture media, among which *Gardnerella vaginalis* stands out. However, 16s rRNA pyrosequencing techniques and the most recently studied biomolecular techniques (multiplex PCR, NAAT and metagenomics), in addition to confirming species already identified by culture media, such as *Prevotella bivia*, *Mobiluncus curtisii* and *Mycoplasma hominis*, also enabled the detection of numerous previously unidentified bacterial species, such as *Atopobium vaginae*, *Leptotrichia sp*., *Megasphaera sp*., *Sneathia sp*., among others.^(38,39)^ Unfortunately, these molecular biology techniques are not yet available for routine clinical care.

The proposed schemes demonstrate similar efficacy in the treatment of BV and can be used during pregnancy.^(40,41)^ Therefore, the route to be used must take into account the patient’s comfort. Note that in pregnant women, if there is no limitation for the oral route, it should be prioritized. The following is a summary of the drugs and doses approved for use:

- Metronidazole 400 mg – orally every 12 hours, for seven days;
- Metronidazole 250 mg – two tablets orally every 12 hours for seven days;
- Metronidazole 0.75% vaginal gel – one applicator (5 g) vaginally for seven days;
- Clindamycin 300 mg – orally every 12 hours, for seven days;
- Clindamycin phosphate 2% vaginal cream – one applicator vaginally (5 g) for seven days.

Metronidazole can be used from the first trimester of pregnancy, even orally, as recommended above. Note that this is a category B drug according to the Food and Drug Administration (FDA) classification. In a cohort study of pregnant women, it was found that even though it crosses the placenta, there was no evidence of teratogenicity or mutagenic effects of metronidazole. With this information, it is considered low risk for pregnant women.^(42)^ Side effects of oral metronidazole include a metallic taste in the mouth, nausea and vomiting.^(41)^ Despite these potential adverse effects, metronidazole is generally well tolerated by pregnant women. Given the potential risk of disulfiram-like effects, abstinence from alcohol during treatment with metronidazole has been advised. However, this guidance is controversial, as the concentration of the aldehyde dehydrogenase enzyme does not change with the use of metronidazole and its derivatives.^(43)^

The treatment of BV during pregnancy has several precise and indisputable indications, including the clinical improvement of the clinical manifestations of the disease, reduction of the risk of sexual dissemination of HIV and improvement of the prognosis of some perinatal parameters.^(41)^ However, the results on the reduction in preterm birth rates are not confirmed,^(44)^ and this is the main argument for not recommending screening for this dysbiosis in low-risk pregnant women.^(40)^

The use of tinidazole is not authorized during pregnancy as it is classified as class C by the FDA. Data from animal studies suggest it poses a moderate risk to the fetus, although human studies are still limited.^(41)^

It is strongly recommended to carry out cure control, which can be done using various techniques, including molecular biology tests and Gram staining, using the criteria by Nugent et al.^(36)^ Studies developed by Sobel et al. (2019)^(45)^ demonstrated that most recurrences result from lack of adherence and correct use of medications, which can be detected by monitoring treatment. In practical terms, treatment control is carried out using a sample of vaginal contents collected, on average, 30 days after the last day of treatment. Direct microscopic examination of the Gram stain sample is used in this evaluation, adopting the criteria by Nugent et al.^(36)^

Although recent studies question the possibility of sexual transmission of BV, to date, concomitant treatment of the sexual partner(s) is not recommended, especially in the first episode.^(40,46,47)^

## How to approach pregnant women with vulvovaginal candidiasis?

Species of the genus *Candida* are considered commensal in humans and can asymptomatically colonize the mucous surfaces of the genital, urinary, respiratory and intestinal tracts, as well as the oral cavity, nails, scalp and skin. However, under certain circumstances, the fungus goes from being an asymptomatic commensal to causing infection. Therefore, candidiasis is considered an opportunistic infection that causes disease depending on the host’s conditions. The immune response and *Lactobacillus* are responsible for maintaining the balance of the vaginal flora, but any change in the environment can lead to fungal proliferation and infection.^(41)^

Candidiasis is known to be a common cause of vulvovaginitis, and approximately 75% of women will experience at least one episode of it in their lifetime. In non-pregnant women, the species of fungus most commonly isolated in cases of vulvovaginitis is *Candida albicans* (70% to 89%), followed by non-*albicans* species such as *Candida glabrata* (4.5% to 20%) and others with lower frequency such as *Candida tropicalis* (2.7% to 7.5%), *Candida parapsilosis* (2.1% to 4.3%) and *Candida krusei* (0.9% to 4.2%).^(48,49)^

A premise based on facts indicates that pregnancy predisposes to vulvovaginal candidiasis, an event that results from hormonal changes, greater local humidity, immunological changes characteristic of the pregnancy state and changes in the vaginal microbiota that alter the pH in that site.^(50)^ Other risk factors are diabetes, immunosuppression, use of glucocorticoids, antibiotics and some personal habits, such as wearing thick fabric clothing (reducing genital ventilation), high-sugar diets and stress.^(48)^

There are controversies regarding the influence of *Candida* vulvovaginitis on gestational prognosis. While some authors have not confirmed this association,^(51)^ others associate candidiasis with preterm labor,^(52,53)^ leaving the discussion about this possible association open.

The clinical picture of fungal vulvovaginitis is mainly characterized by itching of varying intensity, and genital discharge, generally whitish. Symptoms such as discomfort and/or pain, dysuria and dyspareunia may also be present, depending on the intensity of the inflammatory process. During gynecological examination, vulvar hyperemia, edema and, eventually, fissures are frequently observed. Note that the genital tract changes characteristic of the gestational period can make symptoms even more uncomfortable.^(4,41)^

Allergic symptoms can manifest themselves in the genital tract as itching and discharge, which is easily confused with candidiasis, including during pregnancy. Sometimes the increase in vaginal discharge occurring during pregnancy as a result of changes in the genital tract, can cause a certain degree of discomfort, mistakenly leading to the suspicion of candidiasis, hence the need to confirm the presence of the fungus to diagnose this condition. A study of Brazilian pregnant women showed the fungal infection is caused by the *albicans* species in most cases, and the percentage of erroneous clinical diagnoses, in which there was no growth of fungus in culture in Sabouraud’s medium, is significant.^(54)^ Cytolytic vaginosis, lichen sclerosus and other vulvar dermatoses are also conditions that can be confused with candidiasis.^(3)^ The speculum examination shows hyperemia of the vaginal mucosa and the presence of whitish vaginal contents in variable quantities with a thick or flaky appearance, generally adhered to the vaginal wall.^(4)^

As previously stated, the clinical diagnosis in pregnancy must always be confirmed by the presence of fungi,^(41)^ and the following resources can be used: 1) vaginal pH is generally below 4.5; 2) high vaginal swab examination, placing a drop of vaginal content and a drop of 10% potassium hydroxide on a glass slide, the presence of pseudohyphae and/or grouped yeasts and blastoconidia is observed; 3) bacterioscopy using Gram staining allows the identification of the fungal elements mentioned above; 4) the culture in specific media (Sabouraud or Nickerson) allows the isolation of the fungus, the identification of its species and the eventual performance of an antifungigram, recommended in recurrent cases.^(4)^ It is important to remember the possibility of diagnosing cytolytic vaginosis, another non-infectious alteration resulting from the reduction in vaginal pH.^(55)^

The wet mount vaginal swab has a sensitivity of 50%-60%, depending on the professional’s experience in reading the slide. Therefore, common sense and clinical need will guide progress in requesting tests. A positive wet mount vaginal swab does not require further investigation. However, if it is negative and there are symptoms, it is recommended to continue the diagnostic process with Gram bacterioscopy. Culture in a specific medium is normally reserved for cases of recurrence and allows the identification of fungal species and the performance of an antifungigram. Tests using molecular biology resources have only been used in research.^(4)^

Non-recurrent candidiasis during pregnancy should preferably be treated with topical imidazole in regimens lasting seven days or more. Among the therapeutic options available, the most commonly used medications are clotrimazole (1% cream for seven days), miconazole (2% cream for seven-14 days) and isoconazole nitrate (1% cream for seven days). If the choice is for shorter treatments (three days with cream or single-dose vaginal ovule), there are creams and ovules with higher concentrations available on the market, but they generally cause a lot of discomfort for the pregnant woman. These may be an option for use in non-pregnant women.^(4)^

There is controversy regarding the safe use of oral antifungals during pregnancy. According to some studies, although oral fluconazole is not the drug of first choice in the treatment of candidiasis during pregnancy, there was no evidence of an increased risk of malformation in fetuses of pregnant women exposed to the drug in the first trimester.^(56)^ However, studies with animals suggest that high doses of fluconazole can be embryotoxic and teratogenic, causing malformations, such as cleft palate, cardiac abnormalities and skeletal abnormalities.^(57,58)^ No protocols in humans based on controlled studies evaluating the use of fluconazole during pregnancy are available so far.

Guidelines for the treatment of non-*albicans Candida* species in pregnant women recommend the use of nystatin (vaginal cream for 14 days). Although the use of boric acid vaginal ovules is quite effective against these fungal species, it is contraindicated in pregnant women^(59)^For *Candida kruse*i, the therapeutic response to topical clotrimazole or miconazole is considered satisfactory.

More than three confirmed episodes of candidiasis in one year are considered recurrence. Recurrent cases of candidiasis are frequently associated with inadequate blood glucose control in pregnant women with some degree of alteration in glucose metabolism. One of the biggest problems in the recurrence of candidiasis in pregnant women is linked to the limitation of oral medications and the limitation in identifying other non-*albicans Candida* species. Once these less frequent species are identified, the use of nystatin cream (for 21 days) can help resolve the problem.^(60)^ Another option is the intermittent prolongation of clotrimazole or miconazole intravaginally three times a week until the end of pregnancy.^(59)^

There is no evidence to support the treatment of asymptomatic male sexual partnership.^(40)^ Men sometimes develop a hypersensitivity reaction to the fungus after sexual intercourse, with a sensation of itching and burning, associated with penile hyperemia. In this case, treatment includes eliminating the fungus from the female genital tract. Men do not benefit from the topical use of imidazole, but in symptomatic cases, low-potency corticosteroids based on 2% hydrocortisone may be prescribed.^(60,61)^

## How to approach pregnant women with trichomoniasis?

Vaginal trichomoniasis is caused by *Trichomonas vaginalis*, a motile, flagellated, facultative anaerobic protozoan considered a sexually transmitted infection. During pregnancy, the prevalence varies (3.9% to 24.6%) depending on the diagnostic method used and the population evaluated.^(62)^ The following factors are strongly associated with this infection: multiple sexual partners, being a sex worker, inconsistent use of condoms with a risky sexual partnership, use of illicit drugs, current or previous history of a sexually transmitted infection and contact with people deprived of their liberty.^(63)^

A symbiotic association between *Trichomonas vaginalis* and certain bacteria, more precisely from the *Mollicutes* class, *Mycoplasma* genus, has been described recently. *Mycoplasma hominis* and *Ureaplasma urealyticum* could interact with the protozoan, enter the amniotic cavity and cause potentially more serious infectious conditions, which could culminate in adverse gestational outcomes such as preterm labor and chorioamnionitis.^(63)^ In this case, the parasite would protect the bacteria recognition by the immune system, in addition to increasing the virulence of both. The expression “Trojan horse” has been used recently to address this association between *Trichomonas vaginalis* and certain *Mollicutes*.^(64,65)^

In addition to gynecological complications involving the occurrence of pelvic inflammatory disease, unfavorable evolution of HPV infection and increased biological vulnerability to HIV,^(66)^ trichomoniasis is also associated with obstetric complications.^(67)^ A recent systematic review and meta-analysis indicated that trichomoniasis is significantly associated with multiple adverse birth outcomes, including preterm birth, low birth weight, and premature rupture of membranes.^(68)^ However, the association of trichomoniasis with some of these complications is not universally accepted.^(69)^

Genital trichomoniasis can be both asymptomatic and symptomatic, which occurs in varying percentages. In practice, the asymptomatic presence of *Trichomonas vaginalis* is detected casually in smears of vaginal contents obtained for other reasons or even in urinary samples. These higher percentages of asymptomatic cases derive from research using molecular biology platforms. The diagnosis of symptomatic cases of trichomoniasis is based on clinical manifestations led by bubbly yellow-green, profuse vaginal discharge of unpleasant odor.^(70)^ This clinical picture often also includes intense vulvar irritation, urethritis and inflammation of Skene’s glands. In some cases of more severe cervical aggression, the ectocervix may appear hyperemic and with red dots. Tissue aggression can be detected with the iodine test (Schiller test) that shows the irregularly stained ectocervix with the characteristic “tiger-striped” appearance at the time of the speculum examination.^(40)^ In general, the vaginal pH will be higher than 5.0 and the amine test, often positive, will characterize the collapse of the common lactobacillary microbiota, giving way to other anaerobic bacterial groups capable of producing gases (bubbles) and volatile bioamines. The basic difference between trichomoniasis and BV is that in trichomoniasis the cervicovaginal epithelium presents inflammatory signs.^(4)^

When present, the clinical findings of trichomoniasis should lead to diagnostic suspicion, which invariably must be confirmed by identifying the protozoan. Direct microscopic examination of the vaginal contents in saline solution constitutes a quick, cost effective, and easy to perform diagnostic resource.^(4)^ However, it has low sensitivity (50% to 65%), with worse performance when used in populations with a low prevalence of this infection. Although this test is not suitable for diagnosing trichomoniasis, unfortunately it is the only one available in many antenatal care services. The presence of *Trichomonas vaginalis* in the type 1 urine test or oncotic cytology indicates the treatment of the pregnant woman and her partner(s), even in asymptomatic cases.^(41)^ In daily practice, the lack of access to tests promotes a favorable field for the practice of syndromic diagnosis,^(40)^despite the recognized and high rate of diagnostic and therapeutic errors.

Culture in a specific medium (Diamond’s liquid medium) has sensitivity between 75% and 96%, but is laborious, expensive and time-consuming to perform (five to seven days for the final result), with no use in daily practice. An interesting alternative, considered the gold standard, is performing nucleic acid amplification tests (NAAT), but access is still restricted in our country.^(41,71)^

Screening during pregnancy has not been recommended as antenatal routine, most likely because of the heterogeneity of methods used in the studies evaluated so far and treatments that are often instituted late, revealing the urgent need for new studies with better scientific design and methodological rigor.^(2,40,41,71)^

Oral metronidazole is preferred in the treatment of vaginal trichomoniasis in pregnant women.^(40,41)^ Although there are indications for single-dose treatment, aiming at improved adherence (single dose of metronidazole 2 g orally), seven-day treatments are more effective.^(71)^ The following regimens can be used: metronidazole 500 mg orally every 12 hours for seven days, or metronidazole 400 mg orally every 12 hours for seven days, which are similarly effective. Single-dose treatment with metronidazole causes more adverse effects, such as a metallic taste in the mouth and nausea/vomiting, with greater intensity. Dosage schedules with metronidazole 500 or 400 mg orally every 12 hours for seven days have the advantage of being better tolerated, as the rate of adverse effects is lower. This prolonged treatment regimen is also indicated in cases of concomitant diagnosis of BV or when dealing with a pregnant woman living with HIV.

Tinidazole or secnidazole should not be used in pregnant women. Since this is a classic sexually transmitted infection, the entire sexual group involved must be treated.^(40)^ The use of metronidazole (2 g single-dose orally) or its derivatives is permitted for partners and for them, single-dose treatments gain in adherence.

The use of barrier methods for sexual activity should be recommended during treatment and up to seven days afterwards. As it is a sexually transmitted infection, the performance of a serological panel concomitantly with tests for hepatitis B and C, syphilis and HIV is recommended.^(71,72)^ Furthermore, remember the importance of microbiological cure control 30 days after the end of treatment.

## Are probiotics indicated for the treatment of vulvovaginitis in pregnant women?

There is still no consensus that the use of probiotics to restore the vaginal microbiota of pregnant women is truly effective during this period and can prevent preterm labor. There is also no consensus on the use of probiotics regarding doses, specificity and their effects on the vaginal microbiota.^(73,74)^ There is no way to expect that the simple replacement of microorganisms without considering host variables will have sustained success.^(75)^ In fact, science still lacks studies supporting the indication of probiotics in the control of vulvovaginitis in general.^(76)^ If the vaginal environment was not conducive to maintaining the original microbiome, its exogenous replacement without acting on factors linked to the host will fuel the failure rates of this strategy.

## Are there advantages to including the partner in antenatal care to control vulvovaginitis?

Including the partner more actively in antenatal care will facilitate the management of vulvovaginitis and allow the development of more comprehensive strategies to prevent these infections, which will consequently improve perinatal health rates. In addition to offering the partners tests for diagnosis and treatment conditions for sexually transmitted infections, their insertion in antenatal care will provide an opportunity for the physician to offer guidance about prophylaxis and the importance of vulvovaginitis in the evolution of pregnancy and the health of the newborn.^(77)^

## Final considerations

Although vaginal infections are the subject of studies whose results guide their correct diagnosis, in practice most gynecologists do not follow these premises and rely only on complaints and physical characteristics of the vaginal contents (syndromic diagnosis). However, these clinical manifestations during pregnancy are not specific and often lead to diagnostic errors. The pregnancy period with its peculiar modifications makes addressing complaints regarding vaginal discharge an additional challenge. The particularities of the vaginal microbiome of pregnant women with a possible infection or dysbiosis in this site are often disregarded.

Adequate investigation of vaginal discharge involves the use of low-cost and easy-to-perform methods, although these are not yet routinely incorporated into antenatal clinics. The trivialization of the diagnosis and the use of polyvalent therapies without specificity contribute to the imbalance of the vaginal microbiota, prolonging the problem indefinitely. Careful anamnesis associated with adequate clinical examination and complementary tests, such as vaginal pH measurement, amine test and fresh Gram-stained bacterioscopy can diagnose the cause of vaginal discharge with a high accuracy rate, enabling its effective management. This would avoid inadequate treatments and maternal and gestational complications, such as prematurity.

In addition to the adequate diagnosis of BV using the criteria of Amsel et al.^(34)^ and the score of Nugent et al.,^(36)^ the treatment of BV with metronidazole for seven days allows excellent cure rates and low recurrence rates, hence, this is the treatment of choice.

Considering fungal infections, the isolated clinical approach to vaginal discharge, in addition to not solving the problem, may cause complications for women’s health. Added to this limitation is the indiscriminate and inappropriate use of antifungals and/or antibiotics, a situation that clearly favors the imbalance of the vaginal microbiota, predisposing to the recurrence of infection, often more serious than the initial episode. The existence of non-infectious problems involving vaginal discharge and self-medication is a frequent situation and can transform vulvovaginal candidiasis into a difficult-to-treat condition. Therefore, microscopic examination, culture and fungigram should (in stages) be part of the diagnostic arsenal for candidiasis, and treatment in pregnant women should be vaginal, using miconazole, clotrimazole or isoconazole. If non-*albicans* candidiasis is identified, nystatin is the therapeutic option. The control of factors predisposing to candidiasis is important.

From a practical point of view, fresh examination is the most used laboratory resource to corroborate the clinical manifestations of genital trichomoniasis. Despite the high sensitivity and specificity of molecular biology tests, they are still not accessible to women who depend on public healthcare in our country. The indicated treatment is metronidazole orally for seven days, reserving its derivatives or even single-dose metronidazole for the mandatory treatment of sexual partnership.

Including men more actively in antenatal care can improve their adherence to treatment and their respect for sexual abstinence during treatment, facilitating the management of vulvovaginitis. This will also allow the development of more comprehensive strategies to prevent these infections and, secondarily, prematurity.
